# The Influence of Climate Parameters on Maintenance of Wind Farms—A Galician Case Study

**DOI:** 10.3390/s21010040

**Published:** 2020-12-23

**Authors:** José A. Orosa, Ángel M. Costa, Diego Vergara, Feliciano Fraguela

**Affiliations:** 1 Department of Energy and Marine Propulsion, University of A Coruña, ETSNyM, Paseo de Ronda 51, 15011 A Coruña, Spain; angel.costa@udc.es (Á.M.C.); feliciano.fraguela@udc.es (F.F.); 2 Department of Mechanical Engineering, Catholic University of Ávila, C/Canteros, s/n, 05005 Avila, Spain; diego.vergara@ucavila.es

**Keywords:** moist air, sensors, wind farm, maintenance, data mining, weather

## Abstract

There are different monitoring procedures in wind farms with two main objectives: (i) to improve energy production by the capability of the national electrical network and (ii) to reduce the stooped hours due to preventive and or corrective maintenance activities. In this sense, different sensors are employed to sample in real-time the working conditions of equipment, the electrical production and the weather conditions. Despite this, just the anemometer measurement can be related to the more important errors of interruption of power regulation and anemometer errors. Both errors are related to gusty winds and contribute to more than 33% of the cost of a wind farm. The present paper reports some mathematical relations between weather and maintenance but there are no extreme values of each variable that let us predict a near failure and its corresponding loss of working hours. To achieve this, statistical analysis identifies the relation between weather variables and errors and different models are obtained. What is more, due to the difficulty and economic implications involving the implementation of complex algorithms and techniques of artificial intelligence, it is still a challenge to optimize this process. Finally, the obtained results show a particular case study that can be extrapolated to other wind farms after different case studies to adjust the model to different weather regions, and serve as a useful tool for weather maintenance.

## 1. Introduction

In 1981, the world’s first wind farm in the United States was launched. From this milestone to the present day, the main pursued objectives, together with the technological progress of the equipment, are the increase in reliability, reduction of costs and, consequently, greater profitability in the production of wind energy. In this line, it is worth highlighting some scientific research that, in recent years, has tried to determine which is the best maintenance strategy to apply in wind farms, pursuing the aforementioned objectives. This shows an analysis of the different types of maintenance that are being carried out in wind farms, comparing and discussing these types with reference to their statistical control and with special emphasis on their effect in reducing costs and stoppages due to breakdowns [1,2].

These studies carried out an important analysis of the influence of the type of maintenance applied in 3 to 6 MW wind turbines on the costs of their life cycle. What is more, clear cost reductions were obtained when maintenance is applied based on the condition compared to the maintenance based on the condition and preventive maintenance or only preventive maintenance. In the same way, an analysis is made of the economic impact that it has on the life cycle of wind turbines, estimated at 20 years. The application of the maintenance based on the condition seeing the not only technical but also economic advantages that brings the implementation of this technique in wind farms [3].

In this same sense, a previous economic study was carried out to determine the parameters that determine the return time of the investment in the implementation of maintenance techniques by condition [4]. This reinforces the theory developed during the last few years that the maintenance technique based on the condition is the one that provides the best results in wind farms.

Within maintenance by condition, different investigations have sought the creation of new more effective algorithms by including new predictive variables. In this sense, it must be highlighted that the analysis of the different effects that occur in wind turbines, depending on the direction and speed of the wind and the possible relations with their faults [5]. What is more, the relationship between the maintenance of wind turbines and the climatic conditions to which they are exposed was demonstrated previously [6], showing the relation between wind speed, the power and the failure rate. At the same time, a previous analysis showed the relationship between failure rate and other environmental factors when there are indications of its existence [7]. In 2013, Byon [8] presented a maintenance model based on the dynamic condition that links the external (meteorology) and internal conditions (operating parameters) with linear decision rules. It suggested that a new approach is necessary since taking together these parameters into account improves the results of the maintenance of wind farms by increasing their efficiency and effectiveness.

Other investigations continued to reaffirm the relationship of weather conditions, failure rate and failure modes [9,10]. In the same way, an initial climatic variable-failure classification, by analysis of the variance, was developed to obtain control algorithms in subsequent research works to predict breakdowns and improve the maintenance systems by condition [11]. Finally, in 2017, a new study showed that failure prediction models based only on the age of wind turbine systems and components can be improved by introducing the climatic conditions to which they are exposed to. This study presents a model focused on the general breakdowns of wind turbines with satisfactory results [12].

As it was shown in previous research works, climatological variables are related to failures in wind turbines [13,14]. As a consequence, the objective of the present research work is to perform an initial analysis of the most frequent failures and breakdowns in wind turbines and the possible associated causes. In a second stage, the objective is to be able to predict states of a high probability of occurrence of breakdowns and failures through meteorological variables to be employed as a control algorithm in applications of maintenance, operation and decision of wind farm locations.

## 2. Materials and Methods

In this section, a description of the materials and methods used in the present study will be carried out. In this sense, we can make a description of the wind farm, find the errors defined by the maintenance personnel and, on the other hand, enumerate the various data mining techniques necessary to develop this research work.

### 2.1. Windfarm and Weather Station Description

The wind farm on which the study was conducted is located in the northwest of Spain, in the autonomous community of Galicia. The wind farm has 37 wind turbines of 660 kilowatts, which gives it a total power of 24.42 megawatts (MW). Each wind turbine has a 700 kVA transformation center with a transformation ratio of 0.69/30 kV. All these transformation centers are connected by underground medium voltage lines for energy evacuation at 20 kV, with the transformer substation 20/132 kV.

The wind turbines of this wind farm have 46 m-diameter rotors. The rotor consists of three upwind blades with a sweeping area of 1662 m^2^. The main axis transmits the power to the generator through the speed increaser which consists in a planetary stage and three-stage parallel axes gearbox from the speed increaser, the power is transmitted to the electric asynchronous generator with two speeds of 1012 rpm and 1517 rpm to deliver a power of 180 and 660 kW, respectively. Finally, it is interesting to highlight, for a future analysis, that the anemometer employed in all these wind turbines consists in a simple cup anemometer due to the resistance of its mechanical elements to a severe weather conditions.

### 2.2. Climatic Variables

Different types of weather stations were employed during this research work. First of all, as it is common in this kind of wind farms, a climatic station placed near the park give us air temperature, relative humidity, wind velocity and direction with a time-frequency of one hour. At the same time, the Environmental Information system of Galicia (SIAM) [15] facilitates those accessing information on the environment and climatology. Another organization that provides the meteorological data is the Forest and Environmental Research Centre of Lourizán (Ministry of Environment of the Xunta de Galicia). This center is made up of 42 stations of climatologic observation, distributed throughout the Galician region. In 1988, it began the installation of a modern network of automatic stations of meteorological observation. In 2000, 23 new stations arrived which transmit information in real-time.

In winter months there are widespread episodes throughout Galicia of high winds, therefore overproduction at the same time in all the parks and therefore the network will have to be regulated. The more parks installed in Galicia, the more problems there will be during those months, and so the network must be taken into account or adapted [16].

### 2.3. Description of the Main Faults Object of Study

Once the climate was defined, the types and frequencies of failures were analyzed with special attention to their economic implications. The first step was to analyze the frequency of each error, and define its percentage of occurrence. The stoppages due to power control and low voltage were the most frequent errors. It can be seen more clearly if we analyze the frequency of each error and determine its percentage concerning the total number of errors, obtaining the following values shown in Table 1. The stop by overproduction (line 1 of Table 1) is a consequence of excessive energy production in the electrical network, which used to happen with high winds. At the same time, anemometer error is related to an overly high speed of the wind turbine anemometer, showing a nonlinear response due to a fluctuation of wind velocity.

Finally, another interesting classification of errors, from the economic point of view, agrees with the percentage of time lost due to them. This new point of view shows that error 4 represents 13.65% of the total stoppage time and error 12 10.42%, and that error 37 is the third, with 10.11% of stoppage time, as we can see in Table 2.

It is interesting to explain nonintuitive errors, such as asymmetric currents SG, circuit breaker differential turn of the nacelle and the low generator (LG) R-phase temperature. In this sense, asymmetric currents SG errors are registered when there is a difference in intensity at the different phases in the connected generator, an emergency stop generally occurs when the difference in intensity between the phase with the highest intensity and the one with the lowest is greater than 40%. This failure is caused by a reduction in the generation of electrical energy, or in the compensation of reactive energy, due to failures in the reference capacitors or thyristors of the electrical production control system of the wind turbine.

Circuit breaker differential turn of nacelle occurs when the programmable logic controller (PLC) input from the nacelle rotation thermals is deactivated. This failure is usually caused by the failure of the nacelle swing motors. Finally, LG R-phase temperature occurs when the measurement of the temperature of the winding of phase R or S of the LG exceeds 140 °C. This error occurs due to damage to the subgenerator or due to an error in reading the PT-100 probes.

### 2.4. Response Surface

A response surface design is a statistical technique which allows to better understand and optimize the responses of the processes [17] and perform a process analysis where one variable is influenced by others. Response surfaces are therefore used to refine the models after determining the more important factors.

The objective of the response surfaces is to determine the mathematical model that best adjusts to the values obtained and thus establishes the values of the variables that optimize the value of the variable of answer [18]. In particular, as one of the most used experimental design methods, the Box-Behnken design is used in this study [19,20] by Minitab software v19 (USA). In this sense, Box-Behnken designs generally have fewer design points than composite central designs and, therefore, are less expensive to execute with the same number of factors. What is more, Box-Behnken designs never include runs where all the factors are at their extreme value, such as all low-configuration values.

The first step in generating a response surface is to determine which variables are influencing the response of interest, as it will be one in accordance with previous research works by means one -way ANOVA analysis. After identifying these variables, the response surface can be determined using a first grade or second-grade model. In the case of the first-degree model, it is created through a first-order polynomial determined by Equations (1) and (2) [21].

(1) $$y=f\left(x_{1},x_{2},\ldots ,x_{i}\right)+ɛ$$

(2) $$y=\beta_{0}+\sum \beta_{i}x_{i}+ɛ$$

where *x*_1_, *x*_2_, ..., *x_i_* are the independent variables, *β*_0_, *β*_1_, ..., *β_i_* are the regression parameters of the estimated surface from the experimental data and ɛ is the experimental error [22].

The first-order model is used when seeking to approximate the response surface over a relatively small region of the independent variable space at a location where there is little curvature in the response function. In most cases, the curvature in the response surface is strong enough that the first-order model cannot define it correctly. In these cases, the use of a second-order model is required [23] by the polynomial indicated in Equation (3).

(3) $$y=\beta_{0}+\overset{k}{\underset{j=1}{\sum }}\beta_{j}x_{j}+\overset{k}{\underset{j=1}{\sum }}\beta_{jj}x_{j}^{2}+\underset{i}{\sum }\overset{k}{\underset{j=2}{\sum }}\beta_{ij}x_{i}x_{j}+ɛ$$

In the case of having a model with two variables, the first-order model would be expressed by Equation (4) and the second-order model by Equation (5).

(4) $$y=\beta_{0}+\beta_{1}x_{1}+\beta_{2}x_{2}+ɛ$$

(5) $$y=\beta_{0}+\beta_{1}x_{1}+\beta_{2}x_{2}+\beta_{11}x_{1}^{2}+\beta_{22}x_{2}^{2}+\beta_{12}x_{1}x_{2}+ɛ$$

In the case of the second-order model, the equation of a response surface gives us the addition of squared terms, unlike an equation of a factorial design, which allow us to model the curvature in the response, which gives us the following advantages [17,23]:

- Mapping a region of a response surface. The response surface equations record how changes in the variables vary response of interest;
- Finding the levels of the variables that optimize a response;
- Selecting operating conditions to meet specifications;
- Being very flexible. It can take a wide variety of functional forms, so it works well as an approximation to the response surface;
- Having considerable practical experience that indicates that second-order models work well to solve real response surface problems.

Finally, due to the interest in response surface, the Minitab software was selected for its calculation in this investigation. Another interesting advantage of the Minitab software is that it allows us to make contour graphs, which allows us to visualize the three-dimensional shape of the response surface and to analyze the levels of the factors in which there is a change in shape or height of the response surface.

## 3. Results

In this section a characterization of the numerical values that define each of the error groups and their possible prediction is given. Given the deterministic impossibility of characterizing these errors, the response surface technique was used, which allowed us to obtain the corresponding multidimensional models for an acceptable level of correlation. These models allow defining a control system that anticipates a failure when approaching climatic conditions. This control technique increases the availability of the park and its corresponding economic benefits.

### 3.1. Weather Conditions

In the present section, a description of the annual climatic variables existing in the park objective of the study is done. In this sense, the descriptive statistical study showed a bimodal wind direction with two more frequent orientation values of 40° and 220° and an average speed of 10 m/s sampled by the weather station placed in the ground as it is in most of wind farms. What is more, these winds appear gusty during the months of November, December and January reaching maximum speeds of up to 50 m/s.

At the same time, the climatic conditions showed an average temperature of about 12 °C and relative humidity of 81%, reaching the highest temperature values during the months of August and September, while the relative humidity reaches its minimum values during the months between February and October. The average climatic variables values obtained in the park objective of the study, are shown in Table 3.

**Figure 1:**
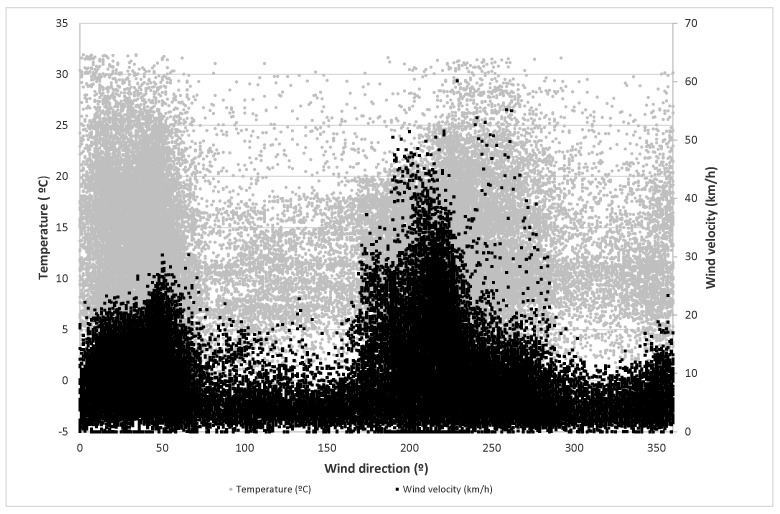
Temperature and air velocity for each wind direction.

**Figure 2:**
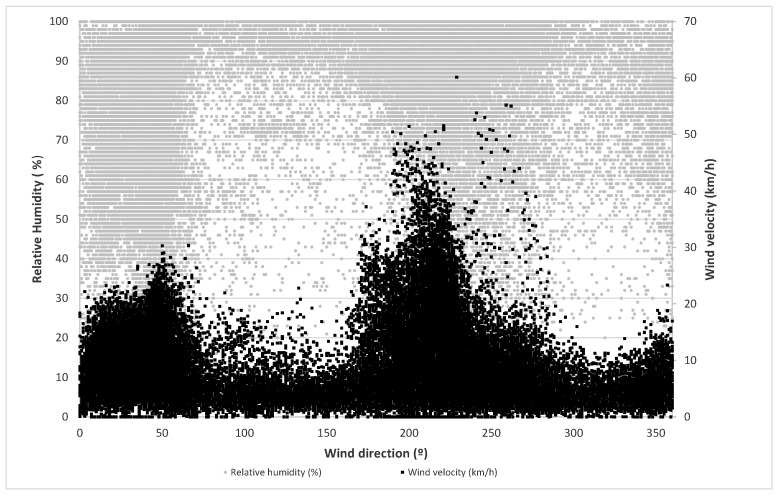
Relative humidity and air velocity for each wind direction.

Finally, the values of temperature, wind velocity and relative humidity versus wind direction are represented in Figure 1 and Figure 2. In these figures, it can be observed that most of the highest velocity values appear at the orientation of 220°. At the same time, most of the highest temperatures and lowest relative humidity occur in the orientation intervals between 0° and 60° and between 200° and 260°.

### 3.2. Errors

In this section, an analysis of the frequency of each failure is carried out as reflected in Table 4. In this sense, it is interesting to highlight that the error 0 is the denomination of a nonerror state and, by consequence, it is associated with the condition of the normal operation of the park. This error 0 reaches 98.1% of the sampled states of the wind farm, as we can see in the first row of Table 4.

### 3.3. Relationship between Variables

In this section, a study of the relationship between the variables is carried out based on a hypothesis test between climatic variables and errors. In previous works, it was possible to determine the existence of a significant relation between errors and climatic conditions at the moment in which they occur. This section is intended to go one step further and to determine the existence of groups of errors based on weather conditions. In this way, as it was explained before, the term error 0 was employed for the conditions existing in the nonfailure period. At the same time, Table 5 defines the climatic conditions existing at the time at which each of the failures occurs with a representative significance value. The significance value was obtained by comparing two variables each time. In particular, Table 5 compares the wind speed with the overproduction error and another study about the wind speed and the high winds error.

### 3.4. Case Study

To be able to relate variables into a model, it is of interest to employ response surfaces which, in our case study, was developed by the Minitab software. After an initial hypothesis test analysis, response surfaces showed that differentiate between the most frequent errors: error 1, error 2 and natural conditions (error 0) for each of the climatic conditions independently or in groups and periods of two months up to one year. In this way, the mathematical models obtained gave us a reduced determination factor. As a consequence, a new point of view was developing defining the minimum wind velocity at which each different error may appear. Finally, the three selected months are from 1 November till 30 January 2010 because they are the months in which the failure is most common.

#### 3.4.1. Modelling and Validation of the Maximum Wind Velocity to Prevent Error 1

As the surface model did not give us adequate models to anticipate the failure, it becomes necessary to apply a new approach to this analysis. After different modelling techniques, it was found that the wind velocity, at the time of each error happens, can be defined as a function of weather variables. By consequence, it is a really useful tool to predict this very expensive cost in a wind farm. What is more, it is possible to model the existing climatic conditions for each of the errors through this statistical tool, as shown in Equation (6) to define the maximum wind velocity at which error 1 probably appears.

(6) $$c_{error\_1}=-\;47.9+\;9.44\;\cdot T_{m\;air}+0.324\cdot \emptyset +0.1455\cdot deg-0.2033\cdot T_{m\;air}{}^{2}+0.00055\cdot \emptyset^{2}-\;0.000767\cdot deg^{2}-\;0.0579\cdot T_{m\;air}\cdot \emptyset +0.00077\cdot T_{m\;air}\cdot deg+0.000746\cdot \emptyset \cdot deg$$

where *Ø* is the relative humidity level (%) and *T_m air_* is the temperature in °C.

Due to this error always appearing at 225°, Equation (6) can be simplified for our case study as:

(7) $$c_{error\_1}=-\;53.99+\;9.613\;\cdot T_{m\;air}+0.4918\cdot \emptyset -0.2033\cdot T_{m\;air}{}^{2}+0.00055\cdot \emptyset^{2}-\;0.0579\cdot T_{m\;air}\cdot \emptyset$$

**Figure 3:**
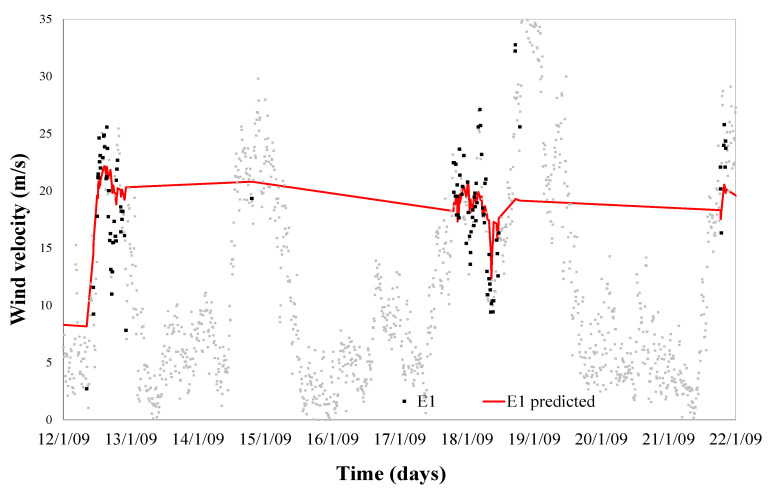
Curve fitting of the predicted limit speed of error 1 versus real errors.

**Figure 4:**
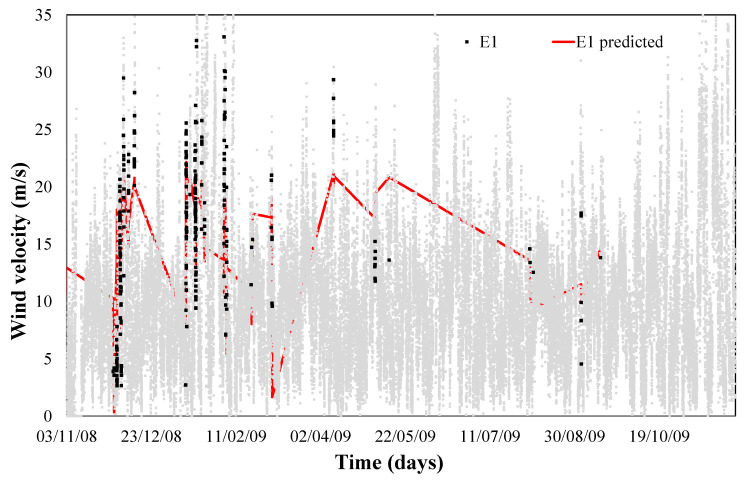
Validation of the predicted limit speed of error 1 versus real errors.

Temperature, relative humidity, and wind direction modelling to predict the limit of wind speed defined for error 1 have a determination factor of 0.92 during January curve fitting; this modelling error can be seen in Equation (6) and shown in Figure 3 and Figure 4.

#### 3.4.2. Modelling Anemometer over Speed (Error 2)

In this section, special attention was employed to analyze the anemometer over speed (error 2), which involves a special interest from a scientific point of view [24]. The main interest for these case studies is to define the wind speed at which wind farm operators must be alerted about a near error risk. In this sense, an analysis about the reliability of this sensor lets us analyze this error at the time in which it most often appears (in the two months’ objective of study from November till March) after training in the months from November till January 2010. From these two figures, it can be observed that anemometer error appears with southwest winds (200°–250°), defined usually by a gusty wind (like with error 1) which reaches a maximum value and, as a consequence, it appears once the main inertia of the sensor is overloaded.

**Figure 5:**
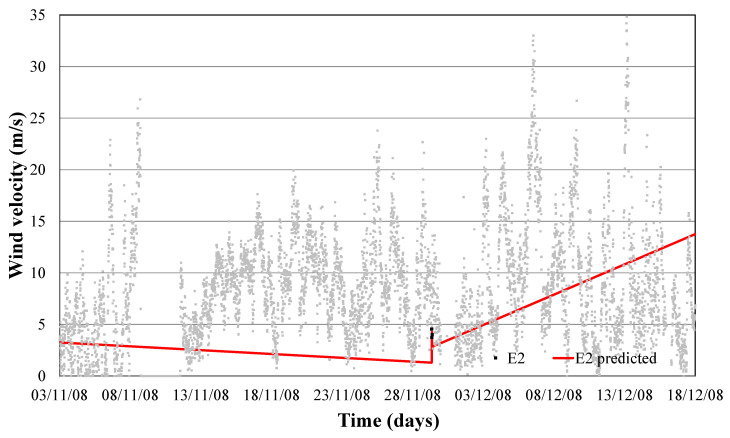
Training error 2 prediction model.

Temperature and wind direction were modelled for the month of January to predict the limit of wind speed at which anemometer failure (error 2) happens. Modeling obtained a monthly determination factor of 0.95. This error modelling can be observed in Equation (8) and shown in Figure 5.

(8) $$c_{error\_2}=17.5\;+\;0.1536\cdot deg-6.07\cdot T_{m\;air}-0.000655\;\cdot deg^{2}+0.399\cdot T_{m\;air}{}^{2}+0.00946\cdot deg\cdot T_{m\;air}$$

Due to wind direction, for our particular case study, the time that an anemometer error appears is about 225°, and we can simplify the maximum wind velocity as a function of temperature to:

(9) $$c_{error\_2}=18.91-3.9415\cdot T_{m\;air}+0.399\cdot T_{m\;air}{}^{2}$$

where *T_m air_* is the average air temperature in the weather station.

**Figure 6:**
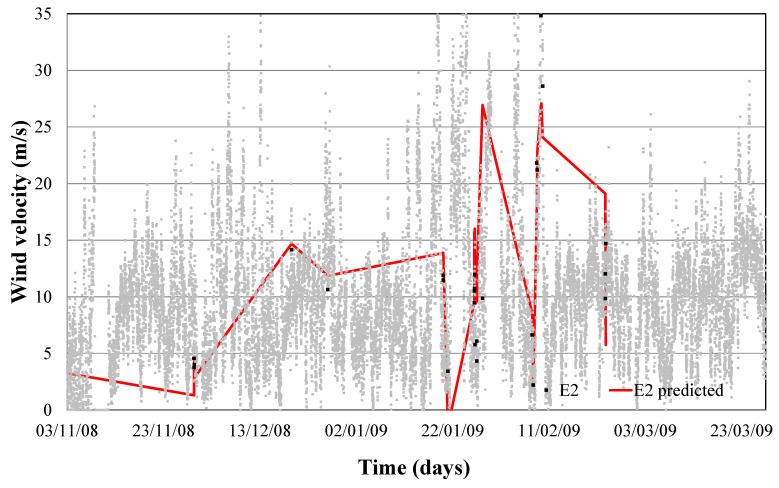
Validation error 2 prediction model.

When this model is tested over a yearly period, a determination factor of 0.65 was obtained, as we can see in Figure 6. Thus, when wind with determined humidity, direction and temperature presents a certain speed, we would enter the risk zone of failure 1 and 2, as shown in Figure 6.

**Figure 7:**
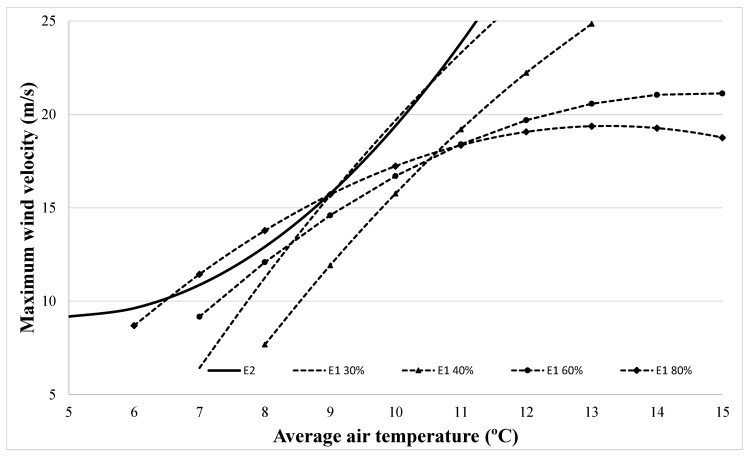
Maximum wind velocity for error 1 (E1) at different relative humidity and error 2 (E2).

As we can see from Equation (7), error 1, which is an external error, is related, in accordance with ANOVA analysis, with all the climatic conditions. If each model is represented to define the minimum wind velocity at which each error may happen at different levels of relative humidity, Figure 7 is obtained.

From this Figure 7, it can be observed that overspeed and overproduction models showed the same behavior to define the minimum air velocity for a temperature range from 5 to 14 °C; just the increase of relative humidity in the air may reduce this minimum velocity. What is more, from these results, we can identify that the anemometer overspeed error is as a particular case of overproduction at low relative humidity levels (30%).

## 4. Discussion

### 4.1. Wind Farm Errors Characterization and Modelling

This research work aims to determine the existence of a relation between the climatic variables that usually occur in a wind farm and the occurrence of the failure at the time each of them happens. This study, despite the fact it was initially analyzed in previous studies [6,11,25], is a more detailed analysis of the relationship between a nonfailure and a failure has been attempted according to each of the climatic variables present at the time of the failure, as shown in Table 5. This table shows the errors that, compared to the nonfailure, show for each of the climate variables a significance level close to 0.000, that is, they are different from the condition’s climate failure. From this table it can be inferred from an analysis of wind speed, that there is a clear difference in speed conditions under normal conditions and at the time error 1 (overproduction) occurs, and with a significance close to zero in error 32 (high winds), as shown in Table 5.

On the other hand, during the study of the wind direction, you can differentiate in Table 6 the time when stop failures occur by error 1 (overproduction), error 2 (anemometer overspeed), error 14 (gearbox temperature), error 22 (LG R-phase temperature), error 32 (high winds) and error 39 (right and left orientation forwarding), and the not failure conditions. Since ambient temperature has clear influences on conditions in the nacelle, we have established through its study a clear differentiation of the conditions when stop failures occur by error 1 (overproduction), error 2 (anemometer over speed), error 5 (LG overproduction), error 9 (emergency stop), error 15 (vibration sensor) and error 17 (excessive time orienting). This is reflected in the data in Table 6.

Finally, relative humidity has a high number of associated faults when it reaches abnormal values. In this way, you can predict stops by error 1 (overproduction), error 3 (low voltage), error 9 (emergency stop), error 17 (excessive orientation time), error 22 (LG R-phase temperature) and error 32 (high winds).

These results are in line with the logical causes of the errors, as excessive wind top speed will cause problems, and the error 32 (high winds) and high wind conditions will present wind production excess that will lead to stops by power regulation. On the other hand, wind direction can lead to the above errors and, in addition, cause problems of transduction of the anemometer and temperature in the gearbox. Similarly, a very low or very high ambient temperature causes the freezing of anemometers or problems in the yaw mechanism causing too much time to orient.

This air mass, as noted above, can be associated with several of the most common errors such as stoppage by power regulation (error 1; 33% of failures) associated with abnormal values of the four climate variables simultaneous direction, high humidity (100%), temperature (30 °C) and speed peaks of 40 km/h (speed of 11.3 m/s). In the same way, the emergency stoppage (error 9; 5.2%) and excessive time orienting (error 17; 2%) are also associated very high humidity and temperature values as a result of the arrival of winds with this orientation.

First of all, it should be noted that, although these very frequent failures are the ones that have the greatest economic effects, you can only detect those associated with the climate and not many others, as they are the problems in the start-up. Indirectly, this evidence supports the study of significance previously conducted to define which climate variables can detect errors. In this way, we can conclude that the chosen location of a wind farm in the design period will determine, through its weather conditions, the most frequent types of breakdowns and their economic costs.

After defining which climate variables have abnormal values at the time of some failures and with the intention of being able to detect these same failures prematurely, we tested to determine the average values of those variables at the time such errors occur, as can be seen in Table 7.

In this Table 7, for example, it can be inferred that error 2 usually appears under average wind speed of 12.4 m/s, a wind direction of 208.7° N, an outdoor temperature of 8.2 °C and relative humidity of 85.2%. However, these average values cannot be used directly as a control algorithm for fault detection in wind farms, considering that each error is a function of climate variables and high deviation standard of each average value.

Unable to apply the above technique, we tested it with the use of control charts to identify the anomalous values of climate variables at the time when each of the linked errors occurs. However, almost none of the sampling values were found to be outside the control limits, set as the average plus/minus three times the standard deviation. Similarly, following the remaining control chart interpretation standards, a series of upstream or downtrend measurements was sought and has not allowed the anticipation of any kind of failure.

From these control charts, we observed that errors do not occur under abnormal weather conditions, and that the same conditions associated with failure also occur during the normal operation of the wind farm. This implies that we should not treat the study as deterministic based on the number of variables we handle, but it should be treated as a probabilistic study. In deterministic models, a good decision is judged according to the results. However, in probabilistic models, the technician is not only concerned with the results, but also with the amount of risk that each decision brings.

At this point, everything points towards a fateful combinatorial of climate variables, which is a new focus in our study. Therefore, there is a need for the use of data mining techniques to obtain interesting results for the maintenance of the wind farm.

In this way, we have used the methodology of response surfaces, which offers us the mathematical models that determine error 0 and 1 and error 0 and 2 depending on the climatic variables and those associated with the results obtained in the previous sections. This technique has been applied with annual and monthly sample values to determine which of the curves offers the best response.

The result has been really high for the huge mass of data, obtaining the following results with a correlation of almost 70%, very high value for annual and real values becoming higher than that shown in a bimonthly modelling. This is due to the smaller influence of failures with respect to normal operating conditions.

Attempting to validate these models with respect to the actual values obtained, we can conclude that, despite some tendency of the model to indicate a value greater than error 0, it does not reach the value 1 or 2. Somehow, the percentage obtained at the time of failure could be associated with the probability of failure at that time.

With this imprecise result obtained, a new approach to response surface analysis has had to be applied, modelling each of the failures based on a combination of climate variables that offer us the speed at which each of the errors would occur, as shown in Equations (6) and (8).

If we now use the limit speed to which each of the faults seems and represent it in a real wind speed diagram, we can define the time when each of the errors occurs, as shown in Figure 4 and Figure 6.

As can be seen, these results fit perfectly at the moment when an air mass with a given temperature, humidity and direction reaches its limit speed and causes the corresponding error.

Figure 4 and Figure 6 confirm that this new approach provides us a reliable control algorithm for detecting time periods where a combination of values of weather conditions (wind speed and direction, outdoor temperature and relative humidity) with a high probability of error.

This new methodology does not seek to predict the exact moment of failure or an estimate of the hours remaining to the occurrence of the fault, but to inform the operators of the wind turbines, at which time of operation there are values of climatic variables that represent an alert for themselves due to a probability of error.

This represents valuable information and a new indicator to take into account in the decision-making of operational actions in wind turbines, in the choice of maintenance strategies to follow in wind farms and in the estimation of the productive capacity of a wind farm in its study phase.

### 4.2. Climate Technical Unavailability

This new indicator will be referred to herein after as “climate technical unavailability”, defined the same as the one where even if there are valid climatic conditions for wind turbines to generate electricity, will not be available because an error occurs.

As mentioned, this indicator can be taken into account in the implementation studies of the new wind farms, to make a more reliable estimate of the annual electricity production that they will develop over their useful life, and therefore a better approximation of the economic profitability of the wind farm that is planned.

The climate technical unavailability of a future wind farm can be calculated with the errors that are related to the climatic variables. In this sense, we can estimate the annual time that the wind turbines, in the location where they are intended to be installed, will be exposed to these critical weather conditions in which the probability of occurrence of each of these errors is presented.

With the estimation of the number of errors by the average stoppage time of each of them, we would have the time of climatic technical unavailability, and, in the same way with the number of errors and the associated cost for repair of each of them, we would have the associated costs and the loss of economic profitability involved.

Taking as an example one of the wind turbines to study and following the state coding of Table 8, we represent in Table 9 the number of minutes that remains in each state.

Using Equation (10) of the overall equipment effectiveness (OEE), its calculation is made by obtaining the OEE of this wind turbine:

(10) $$OEE=\frac{\left(B+C+D+E+F+G+H+I+J+K+L+M+N\right)-\left(C+D+E+F+H+J+K\right)}{\left(B+C+D+E+F+G+H+I+J+K+L+M+N\right)}$$

Therefore, according to Equation (10), this wind turbine has an OEE of 97.17%. Assuming that the proposed methodology in the design phase had been applied to this wind turbine, the time of unavailability due to errors 1 and 2 whose linking has been demonstrated in this work could have been estimated. Considering that error 1 corresponds to the time encoded as H and error 2 is a percentage of the time encoded as F, we would have a stoppage time that implies a loss of availability of 0.7%. Being able to predict a not inconsiderable reduction of 0.7% gives an idea of the possibilities of the application of the control algorithm indicated in the present research paper.

On the other hand, error 1 occurs when the production of electricity exceeds the evacuation capacity of the point to which the wind farm is connected, that is, when there is an excess of production of the different wind farms connected to the same evacuation line. Figure 3 and Figure 4 show that we can determine in what climatic conditions there is the probability of occurrence of such overproduction, and, in the same way, if we are outside those conditions, that probability does not exist. Therefore, if you could modify the value of any of the climate variables, you could eliminate the combinatorial of variables that cause the error, while a priori this does not seem possible, if it is.

In order to modify the values of the variables, you can act on the value of the wind speed by modifying the angle of incidence of the wind turbine blades which would amount to operating with lower wind speed, and thus away from the risk-of-error conditions. Therefore, as indicated, this methodology can be used in the decision-making of operational actions.

When older, this action would have the immediate effect of the increase of the OEE of the wind farm when it is substantially reduced as there would be no downtime due to error 1. However, although increased availability has a double effect on electricity production, on the one hand, electricity production increases during the hours we have increased availability, on the other hand, and we see reduced electricity production when the error conditions are given as lower wind speed is used to avoid the probability of such an error.

Similarly, you can act on the outside temperature in error 2. This error largely associated with the freezing of the anemometer has been eliminated in many wind farms by installing heat resistors thus modifying the outside temperature of the same. In this way, the value of one of the climatic variables has been modified by eliminating the combinatorial of variables that cause the error, which somewhat validates this methodology. Finally, as noted, this indicator is useful in choosing the maintenance strategies to be followed in wind farms.

The action of the variation of the blade angle not only has the effect on the elimination of error 1, but avoids the stops in full production caused by this error that subject the wind turbine components to stresses that can cause over time breakdowns in them. Therefore, it has a self-protection effect on wind turbines that will lead to a lower incidence of breakdowns over time, an increase in mean time between failure (MTBF) and greater durability of the equipment. This will also have a direct link in the wind farm’s life cycle, increasing their useful life and reducing the costs of operating the wind farm. On the other hand, at the moment of weather conditions where there is a probability of error, we can establish preventive maintenance actions aimed at avoiding it. In error 2, for example, greasing and cleaning of the anemometers could be performed to prevent their blockage.

Both preventive maintenance work on the different components of wind turbines and corrective maintenance work to repair faults that do not involve wind turbine shutdown are carried out whenever possible in periods of calm, i.e., where sufficient wind speeds are not given for electricity generation. In the case of having to be performed in productive periods, these works could be performed when the combination of values of weather conditions means there is a high probability of error. In this way, the unavailability generated with preventive or corrective maintenance work could be compensated with the possible unavailability in the event of the error associated with those conditions.

Everything above gives us an idea of the importance of this new approach in the study of the influence of climate variables on the occurrence of errors and the multiple advantages it brings us.

### 4.3. Wind Farm Weather Station Anemometer Improvements

This section shows indications about the measurement of wind turbine anemometer and how its applications of real-time maintenance can be improved by considering usually neglected moist-air psychrometric variables. In this sense, as it was explained before, we did not observe any kind of relation between error 1 condition and the moment of the day or particular extreme values of each related variables, despite the fact it was related by ANOVA analysis. As a consequence, it is just a combination of variables, in agreement with the obtained models, which cause each error. What is more, these new models let us sample weather conditions from wind farm weather stations and define the minimum wind velocity, at the weather station level at which there is probably a wind farm overproduction or a wind turbine anemometer overspeed error.

From Figure 7, it can be observed that wind turbine anemometer overspeed occurs at an average low weather station wind velocity of 10 m/s and 5 °C. Despite this, it is not a representative value due to the gusty wind is the main expected responsible for this error. In this sense, different research studies [26] showed a clear interest in defining the cup anemometers’ measurement error as a function of the gusty wind and its related third harmonic. It is, in a three-cup anemometer, the angular velocity that can be defined as a constant value and harmonic terms. From these harmonics, the third harmonic element exerts a higher influence over measurement, due to the wind acceleration which is transformed into the torque received in the three-cup anemometer three times per revolution. As a consequence, the solution to improve this anemometer is to reduce this third harmonic term. To reach this objective, Pindado et al. [26,27] showed experimental cup centers closer to the rotor axis and smaller cups to give a lower third term harmonic. As a consequence, some recommendations must be considered at the time to define an anemometer for wind farm applications [28]. Based on the results showed in previous sections, it is possible to define mathematical models to predict these errors and to increase the accuracy of the typical cup anemometer measurement by considering not usually employed variables like moist air temperature and relative humidity.

## 5. Conclusions

The present research paper shows an innovative way to analyze the operation and maintenance of a wind farm based on climate changes, according to a complete study of the analysis of the variance of the most representative failures in wind farm systems and weather conditions.

In addition, a new procedure has been developed and validated to identify the most important errors of a wind farm before they occur: overproduction and anemometer over speed error. This procedure is centered on a model and defines the maximum wind speed at which each error is really probable considering variables not usually employed in wind farm operation criteria like temperature and relative humidity, so it can be a really useful tool for real-time operating decisions, reducing economic costs and working times.

## Figures and Tables

**Table 1:** Errors with the more important percentage of appearance.

Code	Error	Percentage %
1	Overproduction	33.0
3	Low tension	15.9
8	Start	9.3
9	Emergency stop	5.2
12	Asymmetric currents SG (Synchronous generator)	3.6
4	Break shoe temperature	3.3
2	Anemometer over speed	2.8
17	Excessive orientation time	2.0
11	Phase sequence error	1.0

**Table 2:** Errors with the more important percentage of time lost respect the total stoppage time.

Code	Error	Percentage %
4	Break shoe temperature	13.7
12	Asymmetric currents SG	10.4
37	Hydraulic central level	10.1
15	Vibration sensor	8.2
9	Emergency stop	7.6
28	Oil level gearbox	7.0

**Table 3:** Annual average values of climatic variables.

Climatic Variables	Average Value
Wind speed (m/s)	10 m/s
Wind direction (°)	40° and 220°
Outdoor temperature (°C)	12 °C
Relative humidity (%)	81%

**Table 4:** Frequency of the main errors of the park during a year.

Error Code	Frequency	Percentage %
0 (no error)	58,822	98.1
1	381	0.6
3	179	0.3
8	105	0.2
10	79	0.1
9	58	0.1
12	41	0.1
4	37	0.1
5	34	0.1
2	32	0.1

**Table 5:** Analysis of the significance of each failure condition for each different climatic variable.

	Error	Error Code	Significance
Wind speed	Overproduction	1	0.000
	High winds	32	0.019
	Overproduction	1	0.000
	Anemometer over speed	2	0.046
	Gearbox temperature	14	0.006
Wind direction	LG R-phase temperature	22	0.000
	High winds	32	0.005
	Right and left orientation forwarding	39	0.043
	Circuit breaker (differential turn) nacelle	45	0.061
	Overproduction	1	0.000
	Anemometer over speed	2	0.000
Temperature	LG overproduction	5	0.090
	Emergency stop	9	0.000
	Vibration sensor	15	0.075
	Excessive orientation time	17	0.000
	Overproduction	1	0.000
	Low tension	3	0.000
Relative humidity	Emergency stop	9	0.002
	Excessive orientation time	17	0.017
	LG R-phase temperature	22	0.099
	High winds	32	0.036

**Table 6:** Summary table of the study of significance by error and climate variable.

Environmental Control Variable	Error Code											
Wind speed	1									32		
Wind direction	1	2				14			22	32	39	45
Temperature	1	2		5	9		15	17				
Relative humidity	1		3		9			17	22	32		

**Table 7:** Example of average values of the climate variables for each error.

Error Code	2	17	4	9	12
Wind speed (m/s)	12.4	8.6	11.4	10.7	8.6
Wind direction (°)	208.7	146.6	144.7	142.6	122.6
Temperature (°C)	8.2	4.9	14.4	18.8	11.6
Relative humidity (%)	85.2	87.5	73.7	70.1	77.9

**Table 8:** Possible operating and error states of a wind turbine.

Code	Description
B	Production (hhh:mm)
C	Corrective maintenance (hhh:mm)
D	Preventive maintenance (hhh:mm)
E	Reforms or modifications (hhh:mm)
F	In error (hhh:mm)
G	High winds (hhh:mm)
H	Overproduction (hhh:mm)
I	Stop by manual order (hhh:mm)
J	Stop by failure of outside equipment (hhh:mm)
K	Stop for external cause (hhh:mm)
L	In calm (hhh:mm)
M	Without communication (hhh:mm)
N	Stop by reset alarm (hhh:mm)

**Table 9:** Minutes in each of the states of a wind turbine.

AE24	B	C	D	E	F	G	H	I	J	K	L	M	N
MINUTES	34,700	138	24	0	804	387	294	28	0	0	8220	0	0

## Data Availability

Data sharing not applicable.
